# Rheumatic Fever: a neglected and underdiagnosed disease. New perspective on diagnosis and prevention

**DOI:** 10.5935/abc.20160150

**Published:** 2016-11

**Authors:** Carlos Eduardo de Barros Branco, Roney Orismar Sampaio, Mario Maia Bracco, Samira Saady Morhy, Marcelo Luiz Campos Vieira, Luiza Guilherme, Luiz Vicente Rizzo, Flavio Tarasoutch

**Affiliations:** 1 Instituto do Coração - InCor, São Paulo, SP - Brazil; 2 Hospital Israelita Albert Einstein, São Paulo, SP - Brazil; 3 Centro de Estudos e Pesquisas Dr. João Amorim (CEJAM) - São Paulo, SP - Brazil

**Keywords:** Rheumatic Fever, Neglected Diseases, Myocarditis, Mitral Valve Stenosis / surgery

The Rheumatic fever (RF) and the rheumatic heart disease (RHD) remain important health problems among populations of developing countries^1^. The number of studies is scarce, and public and private investments are practically inexistent. In addition, the cases of rheumatic fever are underdiagnosed, and are recognized only following permanent valve damage through the manifestation of carditis, more frequently represented by mitral stenosis.^2^

In high-income countries, a tonsillitis is only a "throat infection". However, in developing countries, or even those of average income, as it is the case of Brazil, it may be a harbinger of rheumatic heart disease, a disease that kills approximately 233,00 to 500,000 of RF/RHD sufferers per year globally.^2^ According to a projection by the World Health Organization (WHO) and the latest census by the Brazilian Institute of Geography and Statistics ("*Instituto Brasileiro de Geografia e Estatística"*, IBGE), it is estimated that annually in Brazil 10 million streptococcal pharygotonsillitis occur, totaling thirty Thousand new RF cases, of which up to half may evolve with cardiac involvement.^3^

The great villain is the streptococcus from the A group (*Streptococcus pyogenes*), which triggers a series of pathological reactions, usually starting with tonsillitis and is followed, in certain cases, by acute rheumatic fever, whose outcome may result in the degeneration of the heart valve.^2^ According to Srinath Redd, the former president of the World Heart Federation (WHF), the rheumatic heart disease is a marker of inequality, social injustice and abandonment of countless populations living in poverty. In the last years, defense groups, including the WHF, are making efforts to rectify this unequality.^4^

The negligence is manifested in the relative lack of engagement in the control of the disease on the behalf of governments, society and fostering agencies. The number of academic publications between 1996 and 2006 were 66% lower than between 1966 and 1976.^5^

The RF was responsible for 5.1 million potential disability-adjusted life years ("DALYs"), resulting from 280,000 deaths, in 2004, and it was the seventh and eighth causes of mortality and morbidity due to neglected diseases, respectively. However, the G-Finder report on research and development for neglected diseases identified only four organizations that invested in this infirmity in the last 4 years.^5^ The funding is disproportionate when compared with the other neglected diseases. Public research institutes contributed with 91.4% of the funds, and philanthropic organizations, with 8.6%. On the other hand, funding from the pharmaceutical industry was insignificant.^5^

Late diagnosis is due to the fact that the stethoscope is the only noninvasive tool available to doctors in remote places of low social and economic level where RF and RHD are more prevalent. However, the detection frequency of rheumatic carditis through cardiac auscultation is usually low, and depends on factors such as the experience of the examiner.^6^ The echodopplercardiogram was shown to be more sensible and specific than cardiac auscultation in the early detection of RF and RHD,^7^ but it is still little available in low-income settings.

Marijon et al.^7^ performed portable echocardiography in schoolchildren in Mozambique and Cambodia and observed a detection rate ten times greater than that from the auscultation. The prevalence of the subclinical carditis then was documented in various studies throughout the world. The greatest prevalence mentioned was among school age children in Togo (3.3%), followed by Mozambique (3.0%), Cambodia (2.2%), Aboriginal Australians (2.2%), Zaire (1.4%) and Namibia (1.2%). The prevalence of 6.7% was estimated in Vietnam.^8^ The echocardiographic criteria for diagnosis of rheumatic carditis were unified by WHF in 2012, aiming to standardize the publications.^9^

The echocardiographic findings are divided into three categories: definitive RHD, borderline RHD and normal^9^. The natural history of the patients deemed as having definitive RHD without previous history of acute and borderline rheumatic fever are uncertain. If they are variants of the rheumatic heart disease, it is not known for certain if the secondary prophylaxis shall prevent the progression of the disease as it occurs with patients diagnosed through the "traditional" method. The false positive cases may have a detrimental impact on the individuals and their families, as well as being a burden for the health system.^10^

The definition of subclinical rheumatic heart disease, according to the WHF, is the functional or structural alteration of the heart to the echocardiogram, consistent with the rheumatic heart disease in the absence of a pathological heart murmur.^11^

The prognostic of the RHD is directly linked to the gravity of the initial carditis and recurrent attacks. Approximately 60% of the patients that develop cardiac insufficiency during the first outbreak will have some valvar disease in the next 10 years. However, even these patients have a great chance of improvement and even regression of the cardiac injury is appropriate secondary prophylaxis is performed.

Most of the patients that are enrolled in health programs are symptomatic individuals with advanced disease, indicating they had silent or undetectable acute RF attacks; therefore, they go undiagnosed in the initial outbreaks.

The cost of performing an echodopplercardiogram and the difficulty to access this procedure makes the disease to be underdiagnosed in its initial stage, progressing towards irreversible valve damages with need for surgery for valve replacement.^12^

However, the expenses generated by assistance to RF and carditis patients in Brazil are much more significant. Thus, in 2007, approximately R$150 million in admissions resulting from the RF were used by the Unified Health System (SUS), in interventional procedures, surgery and percutaneous valvotomy due to cardiac sequelae from the RF^3^.

Additionally, the difficulty to diagnose the acute RF is due to various other factors, such as the absence of specific laboratory markers, atypical displays of articular involvement, little availability of echodopplercardiogram in areas where it is most needed, as well as the low sensibility of the Jones criteria. They all contribute towards the underdiagnosis of this pathology^13^.

The appearance of echocardiographic screening programs revealed that the rheumatic heart disease might be considerably more prevalent than imagined among school-aged children in developing countries. These epidemiological evidences show a clear relation between the incidence of acute RF fever and RHD. However, there are few reliable estimations on the incidence and prevalence of acute Rheumatic Fever in developing countries, including in Brazil. The high prevalence of rheumatic heart disease mentioned in various studies leads us to speculate that the incidence of acute RF is much greater than what was previously estimated^14^.

The framework presented above pictures the need to create governmental programs with support from private initiative to establish the prevalence of the rheumatic fever in our population and develop study and combat groups against this devastating pathology. This initiative is being undertaken in Australia, with international support from the World Heart Federation and the World Health Organization with great success.

In this manner, we shall begin a study to assess the rheumatic carditis and subclinical rheumatic heart disease, in a representative probability sample with children from 5 to 15 years of age, living in the outskirts of the southern region of the city of São Paulo, in a partnership between the Heart Institute from the Clinical Hospital of the University of São Paulo (*"Instituto do Coração do Hospital das Clínicas da Universidade de São Paulo"*, InCor), the Institute of Education and Research from the Albert Einstein Israelite Hospital (*"Instituto de Ensino e Pesquisa do Hospital Israelita Albert Einstein*") and the center of studies and research Dr. João Amorim (CEJAM). In this study, the children shall be clinically assessed, and evaluated by portable echocardiography in accordance with criteria by the WHF. Three groups shall be selected: control (without evidence of rheumatic activity), possible rheumatic activity ('borderline') and definitive rheumatic activity. Genetic studies, HLA analysis and/or immunologic susceptibility shall be compared in the three groups, in order to identify what is the susceptibility standard for the development of rheumatic disease following infection by the *Streptococcus pyogenes*.

Finally, the vaccine for rheumatic fever is under development in certain countries^15^. Brazil contributes with the possibility of a vaccine through development studies of the specific peptide, in execution for approximately 20 years at InCor, which, in turn, resulted in a clinical study of phase I/Iia, starting in 2017. The success of a vaccine in this scenario represents a great hope for our less privileged population.
